# Stress, Emotional Intelligence and the Intention to Use Cannabis in Spanish Adolescents: Influence of COVID-19 Confinement

**DOI:** 10.3389/fpsyg.2020.582578

**Published:** 2020-12-11

**Authors:** Cristina Liébana-Presa, María Cristina Martínez-Fernández, José Alberto Benítez-Andrades, Elena Fernández-Martínez, Pilar Marqués-Sánchez, Isaías García-Rodríguez

**Affiliations:** ^1^SALBIS Research Group, Faculty of Health Sciences, University of León, León, Spain; ^2^Faculty of Health Sciences, Universidad de León, León, Spain; ^3^SALBIS Research Group, Department of Electric, Systems and Automatics Engineering, University of León, León, Spain; ^4^SECOMUCI Research Groups, León, Spain; ^5^Department of Electric, Systems and Automatics Engineering, Universidad de León, León, Spain

**Keywords:** stress, emotional intelligence, cannabis, teenagers, adolescents, confinement, SARS-CoV-2, COVID-19

## Abstract

The disease brought about by the SARS-CoV-2, COVID-19 coronavirus has had an unprecedented global impact. Confinement to control the outbreak may have mental health consequences for the most vulnerable in the population, including adolescents. This study aims to describe and analyze the relationships between the stress variables, Emotional Intelligence and the intention to use cannabis in healthy adolescents, before and after the end of the COVID-19 pandemic containment stage. A comparative correlational study was carried out with validated self-completed questionnaires through an online platform. The sample is made up of adolescents (*n* = 300) aged 13–17 from two different schools in Ponferrada (León, Spain). The analysis of correlation and differences between the groups indicate that confinement has had effects on the mental health of the adolescents, specifically on the emotional manifestations of stress. Furthermore, significant gender differences were found for stress values and Emotional Intelligence. However, no differences have been found for cannabis use intention.

## Introduction

In late 2019, a virus outbreak led to a previously unknown disease called COVID-19, which subsequently spread worldwide. There are at present more than 200 countries and more than two million people infected (Chinazzi et al., 2020). The most common symptoms of this disease include: fever, dry cough and tiredness. Some infected people have only minor symptoms. Most of them (about 80%) recover from the disease without needing hospital care. About 1 in 5 people who get COVID-19 eventually develop a severe condition and experience breathing difficulties. Older people and those with previous health conditions are more likely to suffer from serious conditions. However, anyone can get COVID-19 and become seriously ill (Organización Mundial de la salud, 2020). In Spain, the pandemic brought about by the COVID-19 virus, forced the government to declare a state of alarm (Royal Decree 463/2020, of March 14), for the management of the health crisis situation (Moncloa, 2020).

Since the activation of the health emergency measures, the outbreak of COVID-19 in Spain began to be controlled in May with the start of the de-escalation stages (see Table 1, which shows the chronology of stages implemented in Castile and Leon, the Autonomous Community to which the region in the study belongs). Population efforts and health resources have been key in this control. Confinement measures included the closure of educational centers in the middle of the academic year without reopening them before the end of the school year, which led to various academic and personal difficulties. In the case of adolescents, this situation has brought about changes in both their psychosocial aspect and academic performance, as well as negatively affecting their mental health as a result of the COVID-19 virus (Liang et al., 2020). The school closures due to the pandemic have affected students (UNESCO Education, 2020). From 15 March to 17 May there were 65 days of confinement for this healthy population. In this context, this research is focused on the well-being of the adolescent population, and to seeks not only to determine whether there is a change, but also to find out how these variables behave: stress, emotional intelligence (EI) and the intention to use substances, in particular cannabis.

**TABLE 1:** De-opening phases in the Autonomous Community of Castilla y León (Spain) and permitted activities (Junta de Castilla y León, 2020a,b; Ministerio de Sanidad, 2020).

**Stage**	**Start date**	**Allowed activities**
Confinement	15/03/2020	Alarm stage: forbidden to travel between regions, teleworking for non-essential jobs. Purchase of basic necessities, assistance to health services and establishments allowed.
Stage 0	04/05/2020	Opening of retail trade with significant restrictions on capacity and rigorous hygiene measures. Special and very restricted hours for people of different age ranges to go out on the street.
Stage 1	17/05/2020	Reopening of premises with limited capacity and preferential opening hours for people over 65. Opening of street markets and terraces with limited capacity. Tourism in nature organized by companies, up to 10 people.
Stage 2	01/06/2020	On-site work in some sectors with 2 m safety distance. It is possible to circulate in groups of maximum 15 people. Different schedules for older people. Opening of retail trade, shopping centers and flea markets with less capacity.
Stage 3	15/06/2020	Larger capacity in bars and restaurants, meetings of max. 20 people. Travel within the province is permitted and time slots are eliminated according to the age of the population. Increased capacity in shops, museums, and sports activities.
New normality	21/06/2020	End of the State of Alarm.

Previous studies have shown that, despite public awareness, confinement causes stress levels that affect quality of life during epidemics (Yuan et al., 2020). Recent research with adolescents (Yang et al., 2020), in Wuhan, shows that there is widespread psychological stress in this population, despite positive behavioral compliance with personal hygiene practices. The literature indicates that effective coping strategies can protect individuals from mental health problems when dealing with emergency situations (Xu and He, 2012). On a general level, the literature to date indicates differences between the sexes in terms of stress levels. Adolescent girls generally have more stressful life events, academic, family, social or friendship, personal, and total stress than teenage boys. However, it is adolescent girls who present higher values of perceived EI (Veytia López et al., 2019). Not only the female gender, but also the student status and physical symptoms are associated with a greater psychological impact of the outbreak, and higher levels of stress, anxiety and depression (Wang et al., 2020). Therefore, students who engage in vigorous physical activity report better mental health and less perceived stress than those who do not (Vankim and Nelson, 2013). In Spain, during to the confinement resulting from the COVID-19 virus, a negative association of previous and current physical activity with stress scores was found in the general population (Planchuelo-Gómez et al., 2020). Furthermore, it is also adolescent girls who perceive and understand emotions better, as well as perceiving greater amounts of stress at a later age (Schoeps et al., 2019). In this sense, EI and perceived self-efficacy seem to play a determining role in stress levels and their satisfactory management in academic contexts, with benefits in both their emotional level (Mascia et al., 2020) and academic performance (Navarro-Mateu et al., 2020). Thus, emotional regulation and general well-being are improved by leading a healthy lifestyle (Lubans et al., 2016). The practice of physical activity has been found to improve personal well-being and disease prevention (Ahn et al., 2016). Students who have a better understanding of their emotions demonstrate higher levels of physical activity (González-Valero et al., 2019). The literature highlights the relationship between physical activity and the different dimensions of EI. In studies focusing on the university population, those young people who were physically active in their free time showed higher levels of EI (Gutiérrez and Araya-Vargas, 2014), and particularly of repair and attention (Acebes-Sánchez et al., 2019). Those college students who exercised regularly and continued their exercise habits during the COVID-19 outbreak correlated significantly with lower scores on the depression, anxiety and stress scale (Deng et al., 2020). However, the confinement brought about by COVID-19 virus reduced the outdoor activities of adolescents, and it has been seen how, although boys are more resistant to social isolation, girls experience a greater impact on their moods (Zhang et al., 2020).

The current situation affects children, adolescents and families so exceptionally. As educational centers were closed, social contacts became greatly limited, and leisure time activities were canceled (Fegert et al., 2020). Measures implemented in the field of physical distancing resulting from the COVID-19 virus have been associated with a decrease in physical activity and well-being, an increase in loneliness and Internet use among adolescents (Munasinghe et al., 2020). Among the mental health problems related to quarantine, we find depression, mood swings, anger, insomnia and emotional exhaustion (Brooks et al., 2020). However, most of the studies published to date focus on the general population, with less research into adolescents. In the face of stressful events, high levels of acceptance and coping strategies, such as low self-blame, can help people to be better prepared to respond to stressful events and facilitate the use of active, problem-centered resolution (Finkelstein-Fox et al., 2019). Thus, adolescence is one of the highest risk stages for both the initiation of substance abuse and its continuance (del Carmen Pérez-Fuentes et al., 2018), during which cannabis use may appear as an escape mechanism from stress (Low et al., 2012).

Cannabis is the third most widespread drug among students aged 14–18 and the most prevalent illegal substance according to the most recent data from the ESTUDES survey (ESTUDES, 2018). Currently, in Spain, 3 out of 10 young people admit to having used cannabis, and the prevalence of those who have used it at some time in their lives is slightly higher in males (34.5%) than in females (31.5%) (ESTUDES, 2018). Cannabis users cope with stress less flexibly (Kruczek, 2017), and also show lower values of emotional repair (Sandín, 2003). The literature indicates how alcohol and tobacco use is related to EI, resilience and family functioning, the latter being both a protective and risk factor, depending on the circumstances (Molero Jurado et al., 2019). Similarly, while a higher level of emotional regulation capacity is related to a lower frequency of substance abuse, a higher level on the adaptability scale has been associated in a study by Kun et al. (2019) with more frequent substance abuse. Evidence from the literature reveals a negative association between cannabis use and adequate physical activity among adolescents in low- and middle-income countries. This study also shows how adolescents who have used cannabis in the past 30 days are less likely to achieve adequate levels of physical activity (Ashdown-Franks et al., 2019).

In order to provide evidence to promote mental health in adolescents, the aim of this study is to describe and analyze the relationships between the variables of stress, EI and intention to use cannabis in healthy adolescents, during and after the end of the confinement stage of the COVID-19 pandemic.

## Materials and Methods

### Study Design

The design of the study is correlational comparative, following the modality of quantitative research without intervention, non-experimental. The technique used in the data collection was the questionnaire.

### Study Sample

Two samples were obtained by convenience, non-probability, and sampling. In order to obtain homogeneous characteristics, they were chosen from adjacent neighborhoods with similar socio-demographic characteristics in the town of Ponferrada (León, Spain). All of the students were enrolled in the 2019/2020 academic year in two Obligatory Secondary Education (ESO) centers. As an inclusion criterion, all students from first to fourth grade were included, with ages ranging from 11 to 17 years old. A total of 216 students from centre 1 and 271 from centre 2 gave their consent to participate through the authorization of their parents or guardians. Finally, 300 adolescents participated in the study.

### Variables and Measurement Instruments

#### Emotional Intelligence

The EI was assessed through the Trait Meta-Mood Scale (Salovey et al., 1995) in the validated Spanish language version (TMMS-24) (Fernández-Berrocal et al., 2004). It consists of 24 items divided into three dimensions of 8 items each: attention (α = 0.90), clarity (α = 0.90) and repair (α = 0.86). Responses are scored using a five-point Likert-type scale (not at all, somewhat, fairly, very, and completely). The analysis of the scale is done taking into account the scores obtained in each of the three dimensions separately (Fernández-Berrocal et al., 2004).

#### Stress

Stress was measured using the Student Stress Inventory-Stress Manifestations (SSI-SM) (Fimian et al., 1989) validated in Spanish for adolescents (Espejo et al., 2011). This questionnaire consists of 22 items, with a five-point Likert-type scale (not at all, rarely, sometimes, often, and totally). These items are distributed in three factors: emotional (α = 0.79), physiological (α = 0.62) and behavioral (α = 0.66).

#### Intention to Use Cannabis

The validated Cannabis Use: Intention Questionnaire-CUIQ was used for the youth population (Lloret et al., 2018). This questionnaire comprises 12 items and four subscales: attitude toward use (α = 0.81), subjective norm (α = 0.70), self-efficacy toward abstinence (α = 0.86), and lastly, intention to use (α = 0.94). Each one of the items is evaluated by means of a Likert type scale from 1 to 5 points.

### Procedure

Data from this study were collected through an online questionnaire. The high schools that wished to participate in the collection of data were selected on the basis of their availability and access by students to the technological means from their homes. The data collection of the number 1 compulsory secondary school was carried out between February 7 and 27, 2020, before the declaration of the state of emergency and, therefore, before the confinement of the adolescents in their homes. In contrast, the data from Compulsory Secondary Education center number two were collected between 11 and 21 May, during the last days of confinement of the adolescents in their homes, at the beginning of the first phase of de-escalation programmed by the government of Spain for the town of Ponferrada.

The on-line questionnaire was carried out in different web programming languages, PHP and MySQL for the dynamic functionality of the same, joined to a front-end based on HTML5, CSS, JavaScript and jQuery, meeting different standards and measurements that facilitate the display of the same on different devices (responsive design). These questionnaires, as well as the database that stores the answers, are hosted on a server, ensuring the anonymization of the procedure from the outset. Moreover, the server in which these questionnaires were hosted has the secure hypertext transfer protocol (HTTPS), which guarantees the confidentiality of the data sent by the different respondents to the server.

### Analysis

The statistical analyses were carried out using the Statistical Package for the Social Sciences software (SPSS v. 26.0). The quantitative variables were expressed with the mean and standard deviation (M ± ST). Qualitative variables were expressed as frequencies and percentages. To assess the relationship between stress, EI subscales and the intention to use cannabis, a correlation analysis was performed using Pearson’s correlations. To determine the existence of significant differences between independent groups, the student t-test was performed. A value of *p* < 0.05 was considered statistically significant.

### Ethical Considerations

The anonymity and confidentiality of the participants in the study was considered at all times. As minors, prior authorization was obtained from their parents or legal guardians to participate in the study, as well as the informed consent of the participants. The data obtained from the research will be treated in accordance with both the Constitional Law 3/2018, of December 5, on the Protection of Personal Data and the guarantee of digital rights and the General Regulation on Data Protection of the European Union EU 2016/679 (RGPD). In addition, permission was requested from each educational center and the competent body in the area of education in the region (Ministry of Education of the Junta de Castilla y León). The study was approved by the ethics committee (ETICA-ULE-035-2019) of the University of León (Spain), which guarantees compliance with ethical and legal issues.

## Results

Table 2 shows the demographic characteristics of the participants in the study from two centers both before and after confinement brought about by the COVID-19 pandemic. The age of the participants ranged from 13 to 17 (14.04 ± 1.043), of which 62% were female and 38% male. The sample before confinement comprised 202 students, and after confinement there were 98. The students were distributed among the 1st to 4th years of Obligatory Secondary Education (ESO) and the majority of the participants were in the 1st and 2nd years of ESO.

**TABLE 2:** Characteristics of the participants.

		Center 1* 202 (67.3%)	Center 2** 98 (32.7%)	**Total 300 (100%)**
Gender *n* (%)	Female	113 (37.7%)	73 (24.3%)	186 (62%)
	Male	89 (29.7%)	25 (8.3%)	114 (38%)
Course *n* (%)	1^o̱^ ESO***	55 (18.3%)	23 (7.7%)	78 (26%)
	2^o̱^ ESO ***	52 (17.3%)	36 (12%)	88 (29.3%)
	3^o̱^ ESO***	48 (16%)	18 (6%)	66 (22%)
	4^o̱^ ESO***	47 (15.7%)	21 (7%)	68 (22.7%)
Age *n* (%)	13 years old	76 (25.3%)	41 (13.7%)	117 (39%)
	14 years old	63 (21%)	26 (8.7%)	89 (29.7%)
	15 years old	49 (16.3%)	15 (5%)	64 (21.3%)
	16 years old	12 (4%)	13 (4.3%)	25 (8.3%)
	17 years old	2 (0.7%)	3 (1%)	5 (1.7%)

*Center 1 Before confinement. **Center 2 after confinement. ***ESO, compulsory secondary education.

The results of this study have shown significant correlations between most of the variables, the majority of which are of a weak and moderate nature both before and after confinement (see Tables 3, 4). Table 3 shows the correlations obtained before confinement resulting from the COVID-19 outbreak took place. There was a strong correlation in stress between the component of physiological and emotional manifestations (*r* = 0.778). In this context, there is a strong correlation between the physiological and emotional stress components (*r* = 0.778). In relation to the intention to use cannabis, the component of attitude toward its use is significantly correlated, although in a moderate way, with stress: emotional manifestations (*r* = 0.260), physiological (*r* = 0.300) and behavioral (*r* = 0.412). As regards EI, the correlation with the attitude toward consumption is given exclusively with the attention component (*r* = 0.238) therefore, we cannot affirm that clarity and reparation have an influence on the attitude toward consumption. In relation to the subjective norm of understanding the perceived pressure to carry out a certain behavior or not, statistically significant correlations of a mild to moderate level appear for all the dimensions of SSI-SM, and the attention and repair of the EI. Emotional clarity is not related to a greater or lesser subjective norm. Self-efficacy toward abstinence correlates with all of the components of stress and EI. Finally, intention to use is significantly correlated, in a mild/moderate way, with all three dimensions of stress and EI attention. Table 4 shows the correlations obtained after the confinement of the second center. The results obtained are very similar to those of the first center; however, the following differences should be noted: There is no correlation between clarity and the emotional manifestations of stress, which may indicate that individuals are not able to understand them. On the other hand, correlations appear between clarity and the physiological and behavioral manifestations of stress, but at a significance level of 0.05, instead of.01 as in the first center. Repair also stands out, which does not correlate with any of the stress dimensions. Attitude toward consumption correlates significantly with behavioral manifestations of stress. Another difference we found with respect to those of the first center is that the attitude toward consumption does not correlate with any of the components of EI. And finally, in relation to the intention to consume it is exclusively correlated with the emotional manifestations of stress. Significant differences were found in the emotional, physiological and behavioral manifestations of stress depending on the sex of the adolescents. We see how women score higher on emotional and physiological manifestations than men, and men score higher on behavioral manifestations than women. In terms of gender differences for EI, it has been identifi ed that the attention component is significantly higher for women and, in contrast, men obtain better results in emotional clarity (see Table 5). Statistically significant differences were found for the emotional manifestations of stress, before and at the end of confinement, it being higher at the end (see Table 6). Results for the intention to use cannabis were above the 50th percentile both of the centers and for the male/female comparison.

**TABLE 3:** Center 1 before confinement.

			**SSI-SM**			**TMMS 24**			**CUIQ**		
**Questionnaire**	**Dimensions**		**1**	**2**	**3**	**4**	**5**	**6**	**7**	**8**	**9**
SSI-SM	1. Emotional	r									
		Sig									
	2. Physiological	r	0.778**								
		Sig	0.000								
	3. Behavioral	r	0.661**	0.665**							
		Sig	0.000	0.000							
TMMS 24	4. Attention	r	0.503**	0.481**	0.362**						
		Sig	0.000	0.000	0.000						
	5. Clarity	r	0.208**	0.253**	0.222**	0.473**					
		Sig	0.003	0.000	0.002	0.000					
	6. Repair	r	0.225**	255**	0.195**	0.469**	0.724**				
		Sig	0.001	0.000	0.006	0.000	0.000				
CUIQ	7. Attitude toward consumption	r	0.260**	0.300**	0.412**	0.238**	0.051	0.107			
		Sig	0.000	0.000	0.000	0.001	0.472	0.130			
	8. Subjective standard	r	0.352**	0.335**	0.344**	0.209**	0.124	0.153*	0.474**		
		Sig	0.000	0.000	0.000	0.003	0.080	0.030	0.000		
	9. Self-efficacy toward abstinence	r	0.391**	0.410**	0.329**	0.426**	0.573**	0.624**	0.125	0.263**	
		Sig	0.000	0.000	0.000	0.000	0.000	0.000	0.076	0.000	
	10. Intention to use cannabis	r	0.395**	0.397**	0.418**	0.240**	0.056	0.081	0.563**	0.520**	0.169*
		Sig	0.000	0.000	0.000	0.001	0.429	0.252	0.000	0.000	0.016

Correlations between stress, EI and intention to use in adolescents. **Pearson’s correlation is significant at the.01 level (bilateral). *Pearson’s correlation is significant at the.05 level (bilateral).

**TABLE 4:** Center 2 at the end of the confinement. Correlations between stress, EI and intention to use in adolescents.

			**SSI-SM**			**TMMS 24**			**CUIQ**		
**Questionnaire**	**Dimensions**		**1**	**2**	**3**	**4**	**5**	**6**	**7**	**8**	**9**
SSI-SM	1. Emotional	r									
		Sig									
	2. Physiological	r	0.738**								
		Sig	0.000								
	3. Behavioral	r	0.702**	0.535**							
		Sig	0.000	0.000							
TMMS 24	4. Attention	r	0.545**	0.530**	0.368**						
		Sig	0.000	0.000	0.000						
	5. Clarity	r	0.149	0.223*	0.246*	0.479**					
		Sig	0.144	0.0247	0.015	0.000					
	6. Repair	r	0.180	0.180	0.135	0.499**	0.671**				
		Sig	0.076	0.076	0.184	0.000	0.000				
CUIQ	7. Attitude toward consumption	r	0.304**	0.269**	0.221*	0.048	−0.047	0.153			
		Sig	0.002	0.008	0.028	0.641	0.646	0.134			
	8. Subjective standard	r	0.374**	0.280**	0.215*	0.198	−0.015	0.222*	0.516**		
		Sig	0.000	0.005	0.033	0.051	0.884	0.028	0.000		
	9. Self-efficacy toward abstinence	r	0.362**	0.332**	0.371**	0.528**	0.591**	0.573**	0.162	0.280**	
		Sig	0.000	0.001	0.000	0.000	0.000	0.000	0.111	0.000	
	10. Intention to use cannabis	r	0.253**	0.196	0.188	0.147	0.013	0.194	0.487**	0.462**	0.242*
		Sig	0.012	0.053	0.064	0.149	0.900	0.056	0.000	0.000	0.017

**Pearson’s correlation is significant at the.01 level (bilateral). *Pearson’s correlation is significant at the.05 level (bilateral).

**TABLE 5:** Differences between males and females for the variables stress, emotional intelligence, and intention to use in adolescents.

**Questionnaires**	**Dimensions**	**Men (M ± SD)**	**Women (M ± SD)**	**Total (M ± SD)**	***t***	***p***
SSI-SM	Emotional	19.95 ± 8.63	23.58 ± 9.51	22.20 ± 9.34	−3.32	0.001
	Physiological	9.10 ± 3.98	10.25 ± 4.42	9.81 ± 4.29	−3.40	0.001
	Behavioral	9.61 ± 4.54	9.53 ± 3.95	9.56 ± 4.18	−2.28	0.023
TMMS 24	Attention	21.12 ± 8.55	24.32 ± 8.76	23.10 ± 8.80	−3.09	0.002
	Clarity	25.04 ± 9.19	22.77 ± 8.11	23.63 ± 8.59	2.23	0.027
	Repair	26.31 ± 9.10	24.67 ± 8.86	25.29 ± 8.97	1.54	0.126
CUIQ	Attitude toward consumption	1.00 ± 0.93	0.958 ± 0.851	0.9757 ± 0.88	0.44	0.664
	Subjective standard	0.94 ± 0.74	1.035 ± 0.702	0.98 ± 0.88	−1.12	0.664
	Self-efficacy toward abstinence	4.39 ± 1.44	4.392 ± 1.355	4.39 ± 0.39	0.003	0.998
	Intention to use cannabis	1.07 ± 0.71	1.265 ± 0.965	1.19 ± 0.88	−1.84	0.067

*M, mean, SD, standard deviation.*

**TABLE 6:** Differences between ESO centers before and after confinement for the variables stress, emotional intelligence, and intention to use in adolescents.

**Questionnaires**	**Dimensions**	**Center 1 before confinement (M ± SD)**	**Center 2 after confinement (M ± SD)**	***t***	***p***
SSI-SM	Emotional	21.02 ± 8.88	24.63 ± 9.83	−3.196	0.002
	Physiological	24.63 ± 9.83	10.15 ± 4.54	−0.955	0.340
	Behavioral	9.38 ± 4.16	9.95 ± 4.21	−1.114	0.266
TMMS 24	Attention	22.72 ± 8.98	23.87 ± 8.43	−1.061	0.289
	Clarity	23.40 ± 8.65	24.13 ± 8.496	−0.701	0.484
	Repair	25.67 ± 9.28	24.52 ± 8.30	1.039	0.300
CUIQ	Attitude toward consumption	0.97 ± 0.94	0.98 ± 0.75	−0.102	0.918
	Subjective standard	0.96 ± 0.751	1.08 ± 0.641	−1.317	0.189
	Self-efficacy toward abstinence	4.29 ± 1.48	4.60 ± 1.15	−1.774	0.077
	Intention to use cannabis	1.20 ± 0.95	1.17 ± 0.715	0.351	0.726

*ESO, compulsory secondary education, M, mean, SD, standard deviation.*

## Discussion

The results of this study show how confinement seems to have had effects on the mental health of adolescents, specifically on stress. There is currently no research focusing on the effects that the confinement brought about by COVID-19 pandemic may have on substance use in adolescents, and it remains to be seen whether these effects will be related in the long term to an increased probability of cannabis use.

In terms of EI, our results indicate that women score significantly higher than men in the attention dimension, and men score lowest in clarity. Research indicates how EI skills develop with age. In relation to gender differences, our results are consistent with those reported in previous studies in which women obtain higher EI scores than men (Billings et al., 2014), specifically in the attention dimension (Martínez-Marín and Martínez-Marín and Martínez, 2019; Veytia López et al., 2019; Vaquero-Diego et al., 2020), while men have higher scores for the clarity component. One of the key characteristics of emotionally intelligent people is that they are experts at regulating their emotions and maintaining the quality of their performance during periods of severe stress (Orak et al., 2016). In this sense, the outbreak brought about by COVID-19 pandemic and the subsequent confinement for epidemiological control is a stressful event for students, many of whom were preparing for their exams due to the end of the academic year. In relation to stress levels, our results show differences in terms of sex, women score higher in emotional and physiological manifestations, however, men score higher in behavioral manifestations. This is corroborated with results from previous studies, in which adolescents with lower levels of emotional regulation have more symptoms of stress, social stress and anxiety (Cejudo et al., 2018). However, it has been seen that the female sex, together with stressful life events can predict mental health problems, compared to those with better EI and cognitive skills (Nyarko et al., 2020). This is a factor to be considered and taken into account, as later in university life the regulation of emotions acts as a mediator in models of stress and life satisfaction (Saklofske et al., 2012). As regards the confinement situation, our results show how the COVID-19 pandemic seems to have had an effect on the health and well-being of adolescents, with significant differences at the end of the confinement in the emotional component of stress. There are a limited number of studies addressing these variables during confinement in the adolescent population. However, similarities have been found to that which was obtained in a study carried out at the Spanish University of Valladolid (Odriozola-González et al., 2020) focusing on the impact of the COVID-19 outbreak on the stress of students and teachers. In this study (Odriozola-González et al., 2020) 50.43% showed a moderate to severe impact due to both the outbreak and the confinement, with higher values of depression, anxiety and stress in students compared to university employees. On the other hand, in China a study (Wang et al., 2020) indicates that 53.8% of the population suffered a moderate to severe psychological impact from the COVID-19 crisis and 8.1% showed moderate to severe stress values. Furthermore, women, being students and having specific physical symptoms were associated with a greater psychological impact of the outbreak and higher levels of stress, anxiety and depression (Wang et al., 2020). In line with our results, Liang et al., 2020 (Liang et al., 2020) points out that in China, in population aged from 14 to 35, 40.4% of the population had a tendency to suffer psychological problems due to the outbreak associated with: having lower secondary education having post-traumatic stress symptoms and using negative coping measures, with women being more likely to show post-traumatic stress symptoms.

As regards the relationship between EI, stress and cannabis use, the findings of the study indicate that self-efficacy toward abstinence is highly correlated with EI in both situations. In line with this, the literature indicates that attitudes toward cannabis use, subjective norms, environmental restrictions and intention to use are positively correlated with each other and with weekly cannabis use (Jalilian et al., 2020). Thus, greater difficulty in stress management and empathy more often predict the use of substances such as alcohol, tobacco and cannabis (Kun et al., 2019). In contrast, characteristics indicating high levels of EI, such as higher self-esteem and problem-solving ability, were associated with a lower weekly cannabis use (Jalilian et al., 2020). Such statements are in line with the results obtained in this article, since we can see how those adolescents who, after confinement, pay such attention to their emotions, are not able to act clearly, or with adequate reparation, in other words, they are not able to regulate their emotions. Conversely, the intention to use cannabis is only correlated, in a significant way, with the emotional manifestations of stress. A study of American adolescents shows that susceptibility to tobacco use is not significantly associated with the initiation of cannabis use only, suggesting that the association between susceptibility to using tobacco and the initiation of cannabis use may be driven by the use of poly-substances in young cannabis users (Silveira et al., 2020). On the other hand, there is a significant number of adolescents who are unsure about the perceived benefits of using cannabis, and that use should be for medical purposes, as well as an association between a low perception of harm from use and a high perception of benefits and lifetime use of cannabis (Modeste and Hamilton, 2019). Understanding the cognitive-emotional and behavioral factors underlying the intent to use cannabis is therefore crucial to the effectiveness of countermeasures such as preventive interventions to avoid or reduce cannabis use (Jalilian et al., 2020). Moreover, the early use of substances, including cannabis, can be slowed down by not encouraging sedentary lifestyles among young people (Williams et al., 2019). In addition, the practice of physical activity (PA) in adolescents is related to their mood, where PA is significantly associated with the different moods of adolescents during the COVID-19 epidemic. It has been shown that the higher the level of PA, the better the mood (Zhang et al., 2020).

### Limitations

Although the present paper makes a major contribution to the study of the effect of confinement on the mental health of adolescents and their intention to use cannabis, limitations must be taken into account. We found a convenience sample, only data from center one before confinement and data from center two after confinement were collected. Therefore, we cannot know exactly the situation of both centers during the two time periods considered in this study, for example, there may be factors that influence the results obtained, such as exams periods.

## Conclusion

The exceptional situation experienced due to the COVID-19 pandemic has brought about a period of confinement with consequences for the emotional state of young people. Thus, at the end of the confinement, adolescents in the town of Ponferrada (León, Spain), are perceived to be more stressed (emotional manifestations) than those students before the confinement. In addition, the variables of stress and EI are related, although weakly, with intention of cannabis use. Gender differences between participants indicate that women present greater physiological and emotional manifestations of stress, as well as greater emotional attention. In contrast, men achieve greater emotional clarity. This highlights the need for increased health education which should be combined with emotional management counseling for healthy and vulnerable adolescents. These results can be applied to both drug prevention programs and substance abuse treatment interventions. Promoting adolescent mental health, through the management of emotional competencies such as EI attention, can influence the management of psychological stress affecting students. As shown in the literature, the practice of physical activity has beneficial effects on adolescent well-being, hence future research focusing on physical activity needs to be carried out to improve EI, stress, and prevent cannabis use.

## Data Availability Statement

The datasets presented in this article are not readily available because the database of this article is part of a doctoral thesis that is in process of elaboration, therefore, it is not possible to publish it at present. Requests to access the datasets should be directed to MM-F, mmartf@unileon.es.

## Ethics Statement

The studies involving human participants were reviewed and approved by the Ethics Committee of the University of León ETHICS-ULE-035-2019. Written informed consent to participate in this study was provided by the participants’ legal guardian/next of kin.

## Author Contributions

CL-P, IG-R, and MM-F: conceptualization. CL-P, MM-F, and IG-R: methodology. JB-A and IG-R: software. CL-P, MM-F, and EF-M: formal analysis. IG-R, MM-F, CL-P, JB-A, PM-S, and EF-M: investigation. JB-A, MM-F, and PM-S: resources. JB-A and CL-P: data curation. MM-F, CL-P, IG-R, EF-M, and JB-A: writing—original draft preparation. CL-P, MM-F, EF-M, JB-A, PM-S, and IG-R: writing—review and editing. CL-P and IG-R: supervision. All authors contributed to the article and approved the submitted version.

## Conflict of Interest

The authors declare that the research was conducted in the absence of any commercial or financial relationships that could be construed as a potential conflict of interest.
